# Characterisation of Polyphenol-Containing Extracts from *Stachys mucronata* and Evaluation of Their Antiradical Activity

**DOI:** 10.3390/medicines5010014

**Published:** 2018-01-27

**Authors:** Spyros Grigorakis, Dimitris P. Makris

**Affiliations:** 1 Food Quality & Chemistry of Natural Products, Mediterranean Agronomic Institute of Chania (M.A.I.Ch.), International Centre for Advanced Mediterranean Agronomic Studies (CIHEAM), P.O. Box 85, 73100 Chania, Greece; grigorakis@maich.gr; 2 Department of Food Technology, Technological Educational Institute (T.E.I.) of Thessaly, N. Temponera Street, 43100 Karditsa, Greece

**Keywords:** antioxidants, Lamiaceae, polyphenols, *Stachys mucronata*

## Abstract

**Background**: The aromatic plant *Stachys mucronata* (Lamiaceae) is endemic to the island of Crete (southern Greece), but as opposed to other native Greek members of this family, this species has never been investigated in the past with regard to its polyphenolic composition and antioxidant potency. **Methods**: Aerial parts of *S. mucronata* were exhaustively extracted and partly fractionated through partition, using *n*-butanol and dichloromethane. **Results**: Following an initial examination, which consisted of estimating the total polyphenol content and the antiradical activity, the *n*-butanol extract was found to be by far the richest in polyphenols, exhibiting much stronger antiradical activity compared with the dichloromethane counterpart. On this basis, the *n*-butanol extract was analysed by liquid chromatography-diode array-mass spectrometry, to tentatively characterise the principal polyphenolic components, which were shown to be flavonol but mainly flavone derivatives. **Conclusions**: The most potent radical-scavenging compounds were detected in the *n*-butanol fraction of the extracts, suggesting that the most active antioxidants in *S. mucronate* are relatively polar. The analyses suggested the major constituents to be derivatives of the flavone luteolin, accompanied by apigenin analogues, as well as flavonol glycosides and chlorogenate conjugates.

## 1. Introduction

Numerous secondary plant metabolites have been proven to possess pharmaceutical properties, and various multidisciplinary approaches have been attempted to open novel opportunities for the production of innovative plant-derived pharmaceuticals. In this direction, several strategies have been developed to integrate the knowledge of medicinal plants into drug design [1]. Out of the enormous diversity of bioactive substances occurring in botanicals, the class of polyphenols appears as a prominent phytochemical family, embracing an outstanding range of compounds with a wide spectrum of biological effects [2]. Thus, over the past few years polyphenols and/or polyphenol-containing botanical extracts have been a subject of intensive examination, pertaining to their isolation, identification, and their health- and medical-related properties [3]. 

The Mediterranean flora exhibits a broad biodiversity including a notably high number of native medicinal and aromatic plants, many of which may have several pharmacological potencies. The island of Crete (southern Greece) in particular is unique among the Mediterranean regions, embracing more than 1700 plant species [4], the polyphenolic composition of which is largely uncharacterised. The Lamiaceae family is a distinct botanical group, which includes several well-studied species, such as *Salvia* and *Origanum*, with powerful antioxidant properties [5]. However, species belonging to *Stachys* are rather scarcely studied. In the framework of recent studies on the polyphenolic composition and antioxidant activity of native Cretan Lamiaceae species [4,6], this investigation was carried out with the aim of partly fractionating extracts from the aerial parts of *Stachys mucronata*, a relatively uncommon member of the Lamiaceae family, and characterising their polyphenolic profile and antiradical activity. 

## 2. Materials and Methods

### 2.1. Chemicals and Reagents

Solvents used for liquid chromatography were of HPLC grade. Hexane, methanol, dichloromethane, *n*-butanol, Folin-Ciocalteu reagent, trolox^®^, 2,2-diphenyl-picrylhydrazyl (DPPH^•^) stable radical, anhydrous magnesium sulphate, and gallic acid were from Sigma-Aldrich (Darmstadt, Germany). Sodium carbonate was from Penta (Prague, Czechia). 

### 2.2. Plant Material

The aerial parts of *Stachys mucronata* (Lamiaceae) were collected and provided by the Mediterranean Plant Conservation Centre (Chania, Greece). The plant material was left to dry in a dark and dry chamber for seven days and then ground in a domestic blender and stored in sealed plastic vessels at room temperature, in the dark.

### 2.3. Sample Preparation and Extraction

An amount of 10.1 g of ground plant material was defatted using the Soxhlet technique with hexane for 6 h. The defatted material was freed from residual hexane at room temperature (23 ± 1 °C) and then extracted overnight with methanol, under continuous stirring at 300 rpm. The mixture was filtered through a paper filter (grade 1, pore size 11 μm) and the clear extract was dried in a rotary evaporator (*T* = 40 °C). The solid residue was dissolved by adding hot water (approximately 90 °C) and then left to cool down to ambient temperature. The aqueous solution was then filtered to remove undissolved material.

### 2.4. Solvent Partition

**Figure 1:**
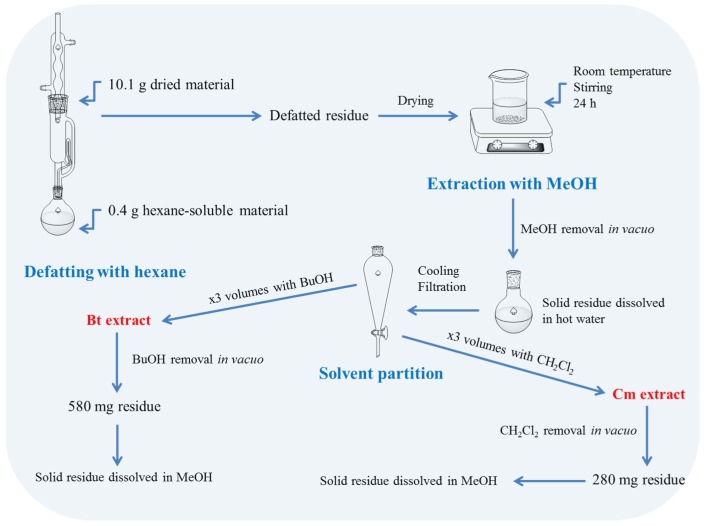
Overview of the analytical procedure followed to generate dichloromethane (Dcm) and *n*-butanol (Bt) fractions of *S. mucronata*.

The aqueous solution (approximately 100 mL) was first partitioned with an equal volume of dichloromethane, and this was repeated three times (3 × 100 mL). The dichloromethane extracts were combined, dried over magnesium sulphate, filtered, and the solvent was removed in vacuo. The solid residue (280 mg) was dissolved in a minimum volume of methanol (usually 2–3 mL) and stored at −20 °C until further analysis. The same procedure was followed with the dichloromethane partition using *n*-butanol, and afforded 580 mg of solid material (Figure 1). 

### 2.5. Total Polyphenol and Antiradical Activity Determination

For total polyphenol determination, a previously reported methodology was used [7]. Results were expressed as milligrams of gallic acid equivalents (GAE) per gram of extract. Antiradical activity was measured using DPPH as the chromophore probe, using a well-established protocol [8]. Results were expressed as mM trolox equivalents (TRE). 

### 2.6. Qualitative Liquid Chromatography-Diode Array-Mass Spectrometry (LC-DAD-MS)

A Finnigan MAT Spectra System P4000 pump was used coupled with a UV6000LP diode array detector and a Finnigan AQA mass spectrometer. Analyses were carried out on an end-capped Superspher RP-18, 125 × 2 mm, 4 µm, column (Merck, Darmstadt, Germany), protected by a guard column packed with the same material, and maintained at 40 °C. Analyses were carried out employing electrospray ionisation (ESI) at the positive ion mode, with acquisition set at 5 and 50 eV, capillary voltage 4 kV, source voltage 25 V, detector voltage 650 V, and probe temperature 400 °C. Eluent (A) and eluent (B) were 2% acetic acid and methanol, respectively. The flow rate was 0.33 mL min^−1^, and the elution programme used was as follows: 0–2 min, 0% B; 2–52 min, 100% B; 60 min, 100% B.

### 2.7. Statistics

All determinations were repeated at least three times and the results were averaged and given with standard deviation. For all analyses, Microsoft Excel^®^ 2010 and SigmaPlot^®^ 12.0 (Systat Software Inc., San Jose, CA, USA) were used. 

## 3. Results and Discussion

### 3.1. Polyphenolic Content and Antiradical Activity

**Figure 2:**
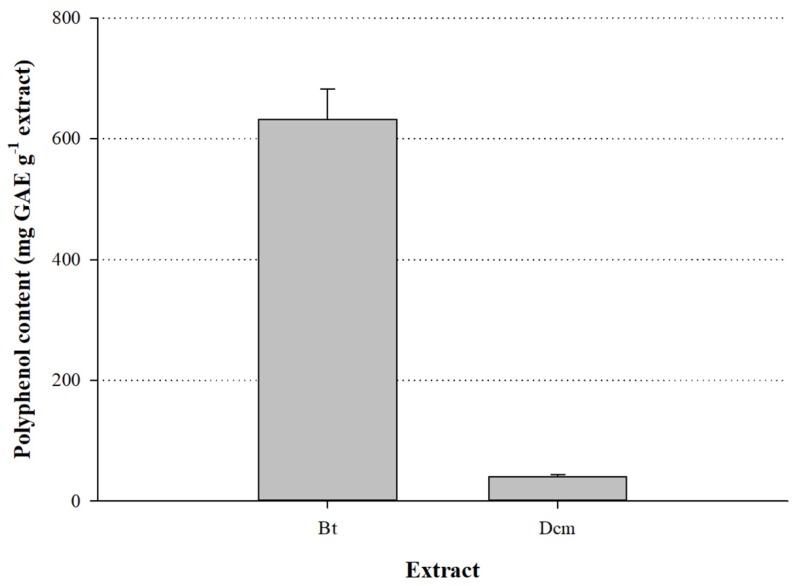
Total polyphenol content of the dichloromethane (Dcm) and *n*-butanol (Bt) fractions of *S. mucronata*. Bars indicate standard deviation.

Extracts were first partly fractionated through partition with dichloromethane and *n*-butanol, to obtain evidence regarding the polarity of the major polyphenols occurring in the aerial parts of *S. mucronate*. As can be seen in Figure 2, the fraction obtained with *n*-butanol (Bt) had a total polyphenol content of 632.0 ± 50.0 mg GAE g^−1^, whereas the dichloromethane (Dcm) fraction displayed a total polyphenol content of 40.0 ± 3.7 mg GAE g^−1^. This finding strongly suggested that the tissue extracted contained relatively polar polyphenols.

**Figure 3:**
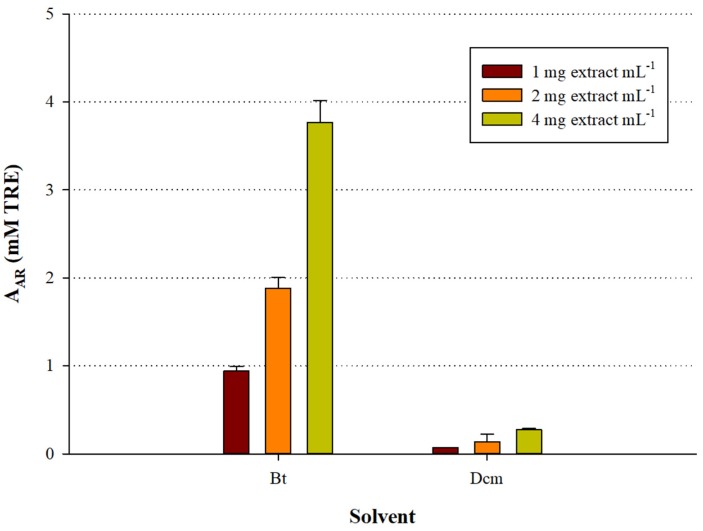
Graph illustrating the A_AR_ of the *S. mucronata* fractions as a function of extract quantity. Bars indicate standard deviation.

As a further step, the extracts were assayed using a representative radical scavenging test (DPPH) to ascertain the presence of antioxidant compounds. Indeed, the results demonstrated that the Bt fraction contained by far more potent antiradical substances compared with the Dcm counterpart (Figure 3). Moreover, the antiradical activity exhibited by both fractions was dose-dependent, showing linear response as a function of total polyphenol concentration (*R*^2^ > 0.98). Based on this outcome, it was concluded that the Bt fraction was particularly enriched in antioxidant polyphenols, and it was chosen for the characterisation of its polyphenolic composition.

### 3.2. Polyphenolic Composition

**Figure 4:**
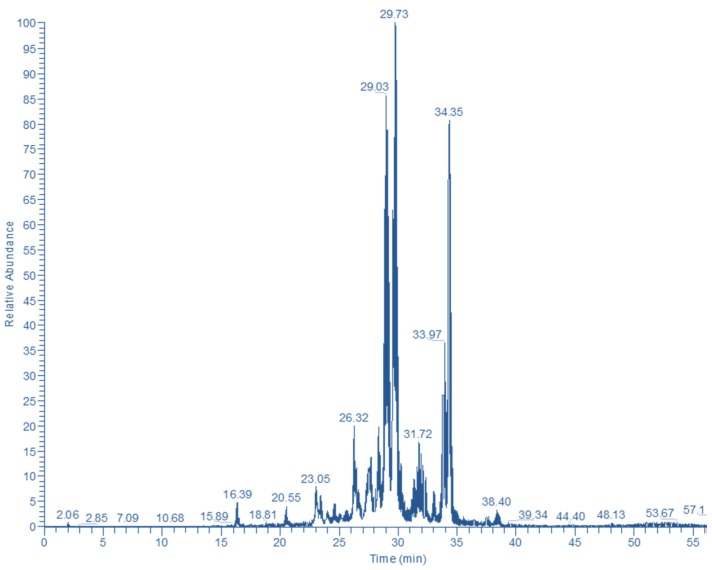
Total ion current (TIC) showing the major polyphenolic phytochemicals detected in *n*-butanol fraction of *S. mucronata*.

The Bt fraction was subjected to LC-DAD-MS analysis to tentatively identify the principal polyphenolic constituents. By obtaining the polyphenolic profile through the total ion current (Figure 4) and the UV-vis spectral characteristics, it was made possible to assign putative structures to 13 compounds (Table 1). Peak #1 displayed a typical hydroxycinnamate UV-vis spectrum, and the diagnostic fragments at *m*/*z* = 355 and 163 (caffeoyl unit) pointed to a chlorogenate derivative [9]. The UV-vis spectrum of peak #2 was consistent with a flavonol structure and the ion at *m*/*z* = 303 suggested a quercetin derivative. Considering the ion at *m*/*z* = 465, this compound might correspond to a substance with a quercetin glucoside or galactoside backbone [9]. Likewise, peak #5 gave a molecular ion at *m*/*z* = 669 and a diagnostic fragment at *m*/*z* = 303, evidencing a hydroxyquercetin acetylallosylglucoside [10].

Peak #3 yielded a molecular ion at *m*/*z* = 449 and a diagnostic fragment at *m*/*z* = 287, suggesting a luteolin glucoside [10]. Peak #6 also gave the ion at *m*/*z* = 449, but the molecular ion at *m*/*z* = 653 along with the UV-vis characteristics indicated the presence of isoscutellarein acetylallosylglucoside [10]. However, peaks #4 and 8 with molecular ions at *m*/*z* = 653 afforded characteristic fragments at *m*/*z* = 287, a finding pointing to luteolin derivatives. In the same line, peak #9 showed a molecular ion at *m*/*z* = 695 and fragments at *m*/*z* = 653 and 287, indicating common structural features. On the other hand, peaks #11 and 12 also gave molecular ions at *m*/*z* = 695, confirmed by their Na^+^ adducts, but yielded fragment ions only at *m*/*z* = 287. 

Finally, peaks #7, 10, and 13 had a common diagnostic fragment at *m*/*z* = 271, evidence of apigenin derivatives. For peak #7, the molecular ion at *m*/*z* = 433 and the UV-vis pattern were in accordance with those reported for apigenin *C*-glucoside [11]. Peak #10 might correspond to apigenin rutinoside [12], but the UV-vis spectral characteristics of peak #13 might indicate a *p*-coumaric acid derivative of apigenin glucoside [10]. 

## 4. Conclusions

In this study, a partial fractionation of *S. mucronata* extracts was carried out in an effort to obtain a polyphenol-enriched fraction. Out of the two fractions generated, the *n*-butanol one showed particularly high polyphenolic content and powerful, dose-dependent antiradical activity. The characterisation of this extract by means of liquid chromatography-diode array-mass spectrometry enabled the tentative identification of 13 polyphenols, which were mainly flavone glycosides, accompanied by flavonol glycosides and a chlorogenic acid derivative. Similar compounds have been detected in several other Lamiaceae species, which possess a variety of beneficial bioactivities. To the best of the authors’ knowledge, this is the first report on the polyphenolic composition of the native Cretan *S. mucronata*, and may provide valuable data for future studies that will aim at investigating the possible biological effects of this particular botanical species, which remain unexamined to date. Since the results from the in vitro examination of the antioxidant activity are only indicative of the antioxidant effects of the extracts, future studies should include both in vitro (e.g., cell lines) and in vivo assays to clearly demonstrate the possible pharmacological potency of *S. mucronata*.

## Figures and Tables

**Table 1:** UV-vis and mass spectral characteristics of the main polyphenolic phytochemicals detected in the Bt extracts of *S. mucronata*.

Peak	Rt (min)	λ_max_ (nm)	[M + H]^+^ (*m/z*)	Other Ions (*m/z*)	Tentative Identity
1	16.39	298 (s), 322	779	765, 751, 731, 503, 489, 457, 355, 163	Chlorogenate derivative
2	24.68	242, 292 (s), 344	763	627, 465, 441, 393, 371, 303	Quercetin derivative
3	25.11	250, 348	449	287	Luteolin glucoside
4	26.32	278, 300 (s), 344	653	611, 287	Luteolin derivative
5	26.52	242, 374 (s), 392	669	653, 517, 303	Quercetin derivative
6	26.72	272, 300, 330	653	449	Isoscutellarein acetylallosylglucoside
7	26.88	265, 340	433	271	Apigenin *C*-glucoside
8	29.03	270, 304 (s), 358	653	287	Luteolin derivative
9	29.73	252, 280, 344	695	653, 287	Luteolin derivative
10	32.74	234, 336	579	447, 271	Apigenin derivative
11	33.73	264, 302 (s), 364	695	717 [M + Na]^+^, 287	Luteolin derivative
12	33.97	232, 276, 306, 332	695	717 [M + Na]^+^, 287	Luteolin derivative
13	34.35	234, 268, 316, 344	579	601 [M + Na]^+^, 271	Apigenin derivative
